# Dosimetric evaluation of adult and paediatric brain tumours planned using mask‐based cobalt‐60 fractionated stereotactic radiotherapy compared to linear accelerator‐based volumetric modulated arc therapy

**DOI:** 10.1002/jmrs.615

**Published:** 2022-10-01

**Authors:** Chin Heng Fong, Robert Heaton, Zhihui Amy Liu, Kecheng Li, Monique van Prooijen, Young‐Bin Cho, David B. Shultz, Derek S. Tsang

**Affiliations:** ^1^ Radiation Medicine Program Princess Margaret Cancer Centre, University Health Network Toronto Ontario Canada; ^2^ Department of Biostatistics Princess Margaret Cancer Centre, University Health Network Toronto Ontario Canada; ^3^ Department of Statistics and Actuarial Science University of Waterloo Waterloo Ontario Canada

**Keywords:** Conformity index, dosimetry, gradient index, icon, mask‐based fractionated stereotactic radiotherapy, volumetric modulated arc therapy

## Abstract

**Introduction:**

We conducted a study to evaluate the dosimetric feasibility of mask‐based cobalt‐60 fractionated stereotactic radiotherapy (mcfSRT) with the Leksell Gamma Knife® Icon™ device.

**Methods:**

Eleven patients with intracranial tumours were selected for this dosimetry study. These patients, previously treated with volumetric arc therapy (VMAT), were re‐planned using mcfSRT. Target volume coverage, conformity/gradient indices, doses to organs at risk and treatment times were compared between the mcfSRT and VMAT plans. Two‐sided paired Wilcoxon signed‐rank test was used to compare differences between the two plans.

**Results:**

The V95 for PTV was similar between fractionated mcfSRT and VMAT (*P* = 0.47). The conformity index and gradient indices were 0.9 and 3.3, respectively, for mcfSRT compared to 0.7 and 4.2, respectively, for VMAT (*P* < 0.001 and 0.004, respectively). The radiation exposure to normal brain was lower for mcfSRT across V10, V25 and V50 compared with VMAT (*P* = 0.007, <0.001 and <0.001, respectively). The median D0.1cc for optic nerve and chiasm as well as the median D50 to the hippocampi were lower for mcfSRT compared to VMAT. Median beam‐on time for mcfSRT was 9.7 min per fraction, compared to 0.9 min for VMAT (*P* = 0.002).

**Conclusion:**

mcfSRT plans achieve equivalent target volume coverage, improved conformity and gradient indices, and reduced radiation doses to organs at risk as compared with VMAT plans. These results suggest superior dosimetric parameters for mcfSRT plans and can form the basis for future prospective studies.

## Graphical abstract

In this comparative dosimetry study, fractionated cobalt‐60 radiosurgery plans (top panels) achieved equivalent target coverage but improved conformity and organ‐at‐risk sparing as compared to VMAT plans (bottom panels). Fractionated radiosurgery may be considered for future prospective study as a highly focused modality for photon radiation of intracranial tumours.

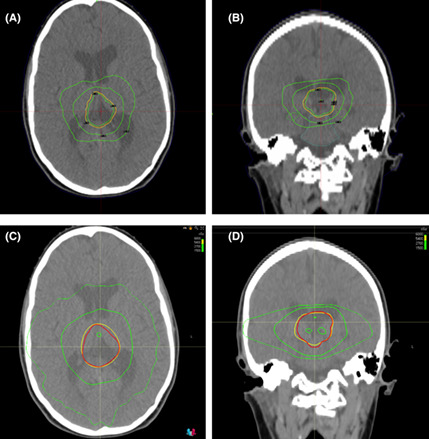

## Introduction

External beam radiotherapy is an integral component in the treatment of many central nervous tumours. Intensity modulated radiotherapy (IMRT) or volumetric modulated arc therapy (VMAT) techniques remain the standard for photon treatment planning and delivery. These techniques achieve good target coverage and high conformity, with a goal to reduce radiation exposure to organs at risk adjacent to the tumour.^1^

Stereotactic radiosurgery (SRS) was originally developed to treat intracranial tumours with high radiation doses in single fractions.^2^, ^3^ This precise, conformal radiation is classically delivered using a Leksell head frame to ensure rigid immobilisation of the cranium during SRS treatment. Nonetheless, the fixation of a Leksell head frame is an invasive procedure requiring the presence of a skilled neurosurgeon, as well as pre‐ and post‐treatment care by a trained nursing team in order to observe for potential complications.

**Figure 1:**
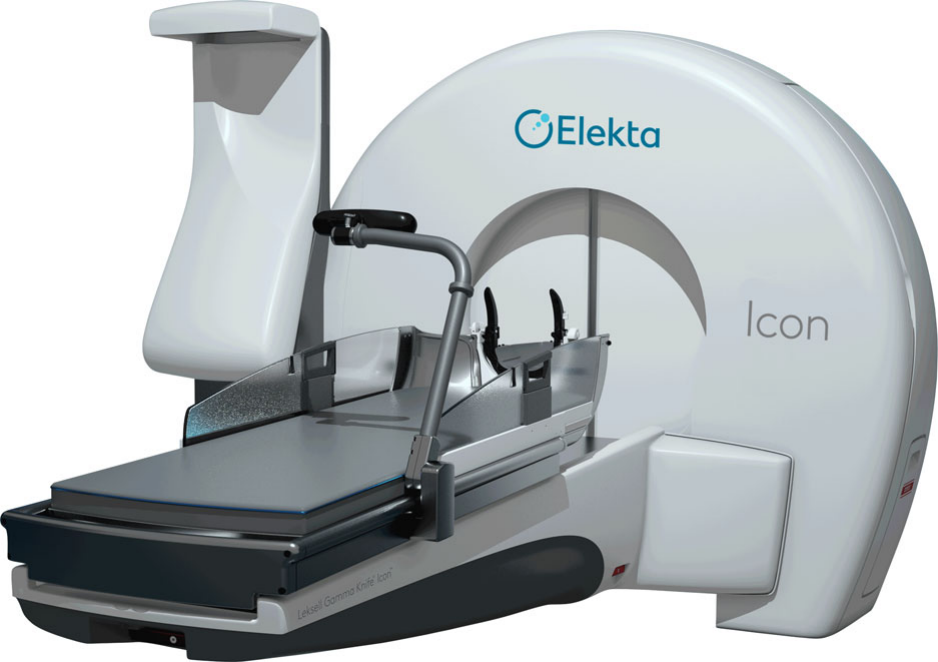
Mask‐based cobalt‐60 fractionated stereotactic radiotherapy (mcfSRT) unit. In this picture, the camera is mounted at the foot of the treatment couch, which monitors a reflective sticker adherent to the patient's nose. Image courtesy of Elekta (link: https://www.elekta.com/company/newsroom/image‐bank/).

There are now commercially available radiosurgery systems that permit cobalt‐60 stereotactic radiosurgery treatment without invasive stereotactic frame placement. An example is Leksell Gamma Knife® Icon™ (Elekta AB, Stockholm, Sweden), which is capable of delivering mask‐based cobalt‐60 fractionated stereotactic radiotherapy (mcfSRT) over multiple treatments, rather than a single treatment. This was made possible using an on‐board cone‐beam CT (CBCT) device, which permits use of a non‐invasive immobilisation technique (thermoplastic mask) while maintaining a high precision of treatment delivery (Fig. 1). The use of a relocatable mask, rather than an invasive frame, improves patient tolerance of the procedure, thus permitting feasibility of radiation treatment over multiple days. A high level of precision is maintained using non‐invasive, infrared camera‐based active position monitoring, which monitors patient position in real‐time and pauses dose delivery if displacement thresholds are exceeded.

In our department, we use the mcfSRT device to deliver single‐fraction SRS and hypofractionated brain treatments, such as for cavity irradiation over 3–5 fractions after brain metastasis resection.^4^ In contrast, linear accelerators are used to deliver conventionally fractionated RT using an IMRT or VMAT technique. However, the question arose as to whether mcfSRT can be used to safely deliver prolonged fractionated treatments over ≥20 fractions, and whether these fractionated stereotactic plans are dosimetrically superior as compared to VMAT plans from a linear accelerator.

Single‐fraction and fractionated SRS share similar benefits with respect to high conformity and steep dose gradients, which makes fractionated SRS an attractive treatment for brain tumours not suitable for single‐fraction treatment. In both children and adults, reducing the dose to organs at risk is of paramount importance to limit the incidence of both acute and late side effects of therapy. Therefore, fractionated SRS may provide dosimetric benefits over fractionated external beam VMAT RT using conventional linear accelerators.

The goal of this study is to evaluate the dosimetric feasibility of mcfSRT by re‐planning patients who have previously received linear accelerator‐based fractionated RT. If mcfSRT plans are dosimetrically superior to linear accelerator‐based RT, then the results of this study will form the groundwork for a prospective study of fractionated SRS for primary brain tumours in both children and adults.

We hypothesised that radiation planning done using the mcfSRT technique is dosimetrically feasible (conformity index near 1, gradient index <3 and coverage >95% with 95% isodose), while statistically significantly reducing the normal brain dose as compared with linear accelerator VMAT radiation plans.

## Materials and Methods

This was a retrospective, in silico dosimetry study. We selected 11 patients with well‐defined, non‐infiltrating, intracranial tumours for the purpose of this dosimetry study. These tumours must be located 2 mm away from critical optic serial organs, including the optic chiasm and left/right optic nerves. Tumours located within brain parenchyma (astrocytoma, oligodendroglioma or glioblastoma) were excluded, because of the lack of evidence of dose escalation in glial neoplasms^5^, ^6^ and higher risk of radionecrosis.^7^ Baseline characteristics of these patients are shown in Table 1. These patients were previously planned and treated with conventional fractionated photon VMAT using Pinnacle 9 (Philips, Amsterdam, the Netherlands) or RayStation 6 (RaySearch Laboratories, Stockholm, Sweden). Patients treated with VMAT were immobilised using a thermoplastic frame and positioned using a pre‐RT cone‐beam CT prior to daily fraction delivery; a 3 mm CTV to PTV geometric expansion was used. Clinical VMAT treatments were planned to achieve at least 95% coverage of the PTV by the 95% isodose line (PTV V95 > 95%). Pre‐defined planning protocols using ring optimisation structures were used, as per institutional standard practice.

**Table 1:** Demographics and baseline characteristics.

Sex	
Female	5
Male	6
Age (years) at time of radiation	
Median	47
Range	12–64
Age category	
Adult	8
Paediatric (age < 18)	3
Diagnosis	
Pituitary adenoma	3
Meningioma	3
Ependymoma	1
Pineal gland tumour	1
Acoustic neuroma	1
Chordoma	1
Medulloblastoma	1
Treatment site	
Supratentorial brain	4
Base of skull	3
Pituitary	3
Internal auditory canal	1
Dose fractionation	
36 Gy in 20 fractions	1
50 Gy in 25 fractions	3
54 Gy in 30 fractions	6
78 Gy in 39 fractions	1
Clinical VMAT plans – Total number of arcs	
One arc (coplanar)	6
Two arcs (one non‐coplanar)	1
Three arcs (two non‐coplanar)	4

These cases were subsequently re‐planned using Leksell GammaPlan® 11 (Elekta AB) for dosimetric evaluation using mcfSRT treatment delivery (Leksell Gamma Knife® Icon™; Elekta AB). Since mcfSRT uses more rigid immobilisation and real‐time position monitoring, a 1 mm PTV expansion from the CTV was used for mcfSRT planning. Our institutional data shows a mean target deviation of 0.34 mm (0.25 mm standard deviation),^8^ further supporting the current practice of 1 mm PTV expansion. The majority of the cases were treated to 50–54 Gy, except for a patient with chordoma who was treated to 78 Gy and one patient with recurrent medulloblastoma who was treated to 36 Gy.

Patient planning data used in this study were prepared and exported from deidentified copies of clinical plans generated in Pinnacle or RayStation. mcfSRT PTV structures were generated in RayStation by applying a 1 mm margin to the CTV. In addition to organ‐at‐risk (OAR) structures, other normal tissue structures were generated through Boolean structure arithmetic. In cases where Boolean structure generation resulted in structures containing an internal cavity region, which is not supported in Leksell GammaPlan, contours on individual image planes were edited to introduce a narrow channel so that the structure perimeter consisted of a single contiguous region on each slice. Once all structures had been constructed, the structure data and the CT image set was transferred to GammaPlan. Dose statistics from the clinical RayStation plans were recalculated on the same set of structures using a uniform 1 mm grid, and dose‐volume histograms (DVH) were exported for analysis.

mcfSRT planning for the target lesions was performed in GammaPlan using the extra fine dose grid (0.5 mm resolution). The Tissue Maximum Ratio (TMR) 10 algorithm was used for dose calculations.^9^ Plans were developed with the goal to cover at least 98% of the mcfSRT PTV with the prescribed dose while respecting critical structure tolerances. In order to limit the maximum dose within the target volume, the prescription isodose was constrained to 70% of the maximum target dose. All plans were generated for a mcfSRT treatment unit with a cobalt‐60 dose‐rate of between 2.9 and 3.0 Gy/min, and all plans were inspected to ensure that individual shot positions were longer than the delivery system's minimum allowed dwell time. DVH data for target and critical structures were exported for analysis using the same software as the clinical RayStation plans. The brain OAR was defined as brain minus the CTV for reporting purposes. Cochlea volumes were small (often <0.1 cc); therefore, D0.1cc was not calculable for this OAR.

The conformity index (CI) is defined by the Radiation Therapy Oncology Group (RTOG) definition as a ratio between the volume covered by the reference isodose (RI), which according to ICRU is isodose of 95%, and the target volume (TV): CI_RTOG_ = V_RI_/TV.^10^ We calculated conformity indices using GammaPlan (for mcfSRT plans) and RayStation (for VMAT plans). The gradient index (GI), defined as the ratio of the volumes of half the prescription isodose to that of the prescription isodose, was used as a dose gradient metric.^11^ For this study, we used the ratio of the volume covered by 47.5% isodose line to that of the volume covered by the 95% isodose line to calculate the gradient index.

Summary statistics were used to describe each variable characterising the mcfSRT and RayStation plans. Comparisons between two plans were made by comparing differences defined as Dose_mcfSRT_ − Dose_VMAT_. Vx represents the volume covered by x% of the prescription dose, unless otherwise specified. Dx represents the dose to x% of the volume, unless otherwise specified. Dmax represents the maximum dose of 1 mm^3^. Two‐sided paired Wilcoxon signed‐rank test was used to compare doses from matched samples. A *P*‐value ≤0.05 was considered as statistically significant. Statistical analyses were carried out using R v3.5.2. This retrospective study was approved by the hospital University Health Network Research Ethics Board (17‐6280).

## Results

A total of 11 patients who were previously planned on RayStation and treated with a linear accelerator with VMAT were selected for this dosimetric comparison study (Table 1). A majority of the selected cases had a treatment site around the base of skull, except for three patients with tumours in brain parenchymal sites (ependymoma, pineal tumour and medulloblastoma).

Our results show that treatments planned using mcfSRT were similar to VMAT with regards to planning target volume coverage (Table 2), but superior in terms of avoidance of organs at risk (Table 3). The volume receiving 95% of the prescription dose (V95) for PTV was similar between fractionated mcfSRT and VMAT, with median of 96.6% and 97.9% respectively (*P* = 0.47). This indicates there was no statistically significant difference in target coverage between mcfSRT and VMAT planning by the 95% isodose line. As expected, the V100 for PTV was higher for mcfSRT with a median of 91.9% versus 38.8% for VMAT and a median difference of 45% (*P* < 0.001). This is because mcfSRT plans had higher doses in the PTV due to the nature of cobalt‐60 SRS planning, which prescribes 70% of the maximum dose.

**Table 2:** Comparison of target dose coverage and treatment details.

Variable	mcfSRT (*n* = 11)	VMAT (*n* = 11)	Difference = mcfSRT − VMAT	*P*‐value
CTV V95				
Median, % [min, max]	99.7 [81.6, 100.0]	100.0 [82.1, 100.0]	−0.3 [−16.6, 0.0]	**0.005**
CTV V100				
Median, % [min, max]	98.4 [74.3, 99.5]	44.4 [22.0, 81.9]	41.7 [9.1, 77.2]	**<0.001**
PTV V95				
Median, % [min, max]	96.6 [75.7, 99.2]	97.9 [71.8, 100.0]	−0.3 [−20.9, 3.9]	0.465
PTV V100				
Median, % [min, max]	91.9 [67.5, 97.1]	38.8 [14.8, 64.0]	45.0 [24.6, 78.7]	**<0.001**
Conformity Index^1^				
Median [min, max]	0.9 [0.8, 1.0]	0.7 [0.6, 0.9]	0.2 [0.0, 0.3]	**<0.001**
Gradient Index^2^				
Median [min, max]	3.3 [2.8, 4.0]	4.2 [3.1, 5.4]	−1.0 [−1.8, −0.2]	**0.004**
Treatment time per fraction				
Median, sec [min, max]	582 [336, 1032]	55 [38, 82]	504 [281, 950]	**0.002**

Bolded values indicate *P* < 0.05.

^1^ A conformity index of 1 is ideal.

^2^ A lower gradient index is better, indicating sharper dose fall‐off.

**Table 3:** Comparison of doses to organs at risk.

Organ at risk	mcfSRT (*n* = 11)	VMAT (*n* = 11)	Difference = mcfSRT − VMAT	*P*‐value
Brain				
V50				
Median, % [min, max]	1.6 [0.1, 3.2]	7.6 [2.2, 18.5]	−5.8 [−15.4, −2.1]	**<0.001**
V25				
Median, % [min, max]	7.0 [0.3, 15.0]	17.7 [9.6, 34.0]	−13.0 [−20.8, −6.7]	**<0.001**
V10				
Median, % [min, max]	27.1 [5.5, 61.0]	36.8 [29.7, 60.1]	−14.3 [−27.5, 4.4]	**0.007**
Mean brain dose				
Median, cGy [min, max]	468.0 [176.0, 993.0]	627.8 [425.9, 1366.0]	−173.7 [−441.8, −47.1]	**<0.001**
Median brain dose				
Median, cGy [min, max]	322.0 [124.0, 785.0]	156.0 [78.0, 1043.0]	2.0 [−461.0, 272.0]	0.966
Maximum brain dose				
Median, cGy [min, max]	7200 [3930, 10,350]	5490 [3598, 7983]	2048 [−88, 3479]	**0.003**
Mean, cGy (SD)	7156 (1960)	5465 (1011)	1691 (1396)	**0.003**
Right optic nerve				
Dmax, median, cGy [min, max]	1960 [70, 5790]	4807 [32, 6098]	−249 [−3015, 106]	**0.014**
D0.1cc, median, cGy [min, max]	1050 [60, 4170]	2706 [29, 5093]	−733 [−2647, 293]	0.054
D50, median, cGy [min, max]	657 [51, 2204]	551 [23, 3283]	63 [−1749, 630]	0.365
Left optic nerve				
Dmax, median, cGy [min, max]	3770 [40, 5570]	4722 [33, 5542]	−342 [−1214, 296]	0.102
D0.1cc, median, cGy [min, max]	1490 [50, 4850]	3451 [29, 5118]	−921 [−2916, 244]	**0.042**
D50, median, cGy [min, max]	702 [39, 3978]	661 [25, 4722]	79 [−1540, 455]	0.638
Optic chiasm				
Dmax, median, cGy [min, max]	3620 [11, 5910]	4989 [65, 6027]	−117 [−3433, 108]	0.067
D0.1cc, median, cGy [min, max]	2900 [60, 4790]	4743 [54, 5455]	−763 [−2590, 158]	**0.014**
D50, median, cGy [min, max]	1348 [50, 4133]	3871 [43, 5165]	−862 [−2781, 212]	**0.014**
Right hippocampus				
Dmax, median, cGy [min, max]	1190 [200, 5080]	3269 [73, 6974]	−845 [−4759, 777]	**0.042**
D0.1cc, median, cGy [min, max]	950 [90, 4200]	1967 [62, 6552]	−847 [−4673, 837]	**0.019**
D50, median, cGy [min, max]	706 [62, 2415]	1185 [34, 4012]	−552 [−2418, 489]	**0.019**
Left hippocampus				
Dmax, median, cGy [min, max]	3380 [200, 6460]	5100 [78, 6063]	−1455 [−2690, 1043]	**0.019**
D0.1cc, median, cGy [min, max]	2610 [170, 5230]	4856 [68, 5465]	−1647 [−2855, 173]	**0.010**
D50, median, cGy [min, max]	1075 [106, 2330]	1637 [41, 3702]	−634 [−1918, 140]	**0.005**
Right cochlea				
Dmax, median, cGy [min, max]	600 [20, 5790]	884 [17, 5231]	−1 [−2441, 1329]	0.520
D50, median, cGy [min, max]	509 [30, 2204]^1^	653 [13, 4547]	−275 [−2992, 201]	0.084
Left cochlea				
Dmax, median, cGy [min, max]	970 [20, 6130]	1587 [14, 5423]	−510 [−1288, 5927]	0.278
D50, median, cGy [min, max]	1452 [48, 4956]^1^	1189 [16, 5347]	−402 [−2025, 4795]	0.106

Bolded values indicate *P* < 0.05.

^1^ Not evaluable for one patient due to small cochlea volume.

The median conformity index was 0.9 for mcfSRT, compared to 0.7 for VMAT (*P* < 0.001), whereas the median gradient index is 3.3 for fSRS and 4.2 for VMAT (*P* = 0.004). This indicated that mcfSRT plans were more conformal and had sharper dose fall‐off than VMAT plans (Table 2). The median beam‐on time was longer for mcfSRT plans, as compared to VMAT plans, by 8.4 min (*P* = 0.002).

**Figure 2:**
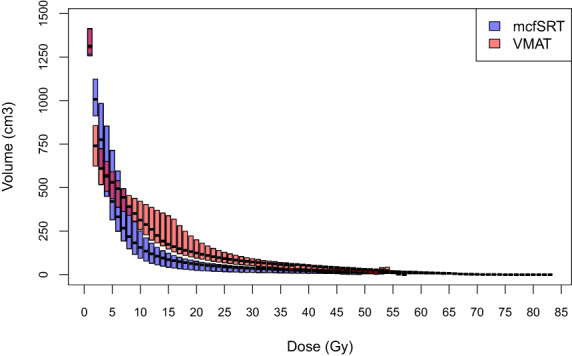
Boxplot comparison of doses to different volumes of the brain, stratified by treatment technique (mcfSRT vs. VMAT). Median doses are shown in a thick horizontal line, while the top and bottom of the coloured boxes denote the 1st and 3rd quartiles. One patient with a 78 Gy prescription was excluded for display purposes.

The radiation exposure to normal brain was lower for mcfSRT compared to VMAT, with brain V50, V25 and V10 showing lower volume of radiation exposure using the mcfSRT technique (Table 3). The mean brain dose for mcfSRT was 25% lower than VMAT (468 cGy vs. 628 cGy), with a median difference of 174 cGy, between the two techniques (*P* < 0.001). However, as expected, the median maximal brain dose was higher in mcfSRT plans compared to VMAT plans (7200 cGy vs. 5490 cGy, *P* = 0.003). A summary of doses to different volumes of brain is presented in Figure 2, demonstrating lower volumes of irradiated brain with mcfSRT.

**Figure 3:**
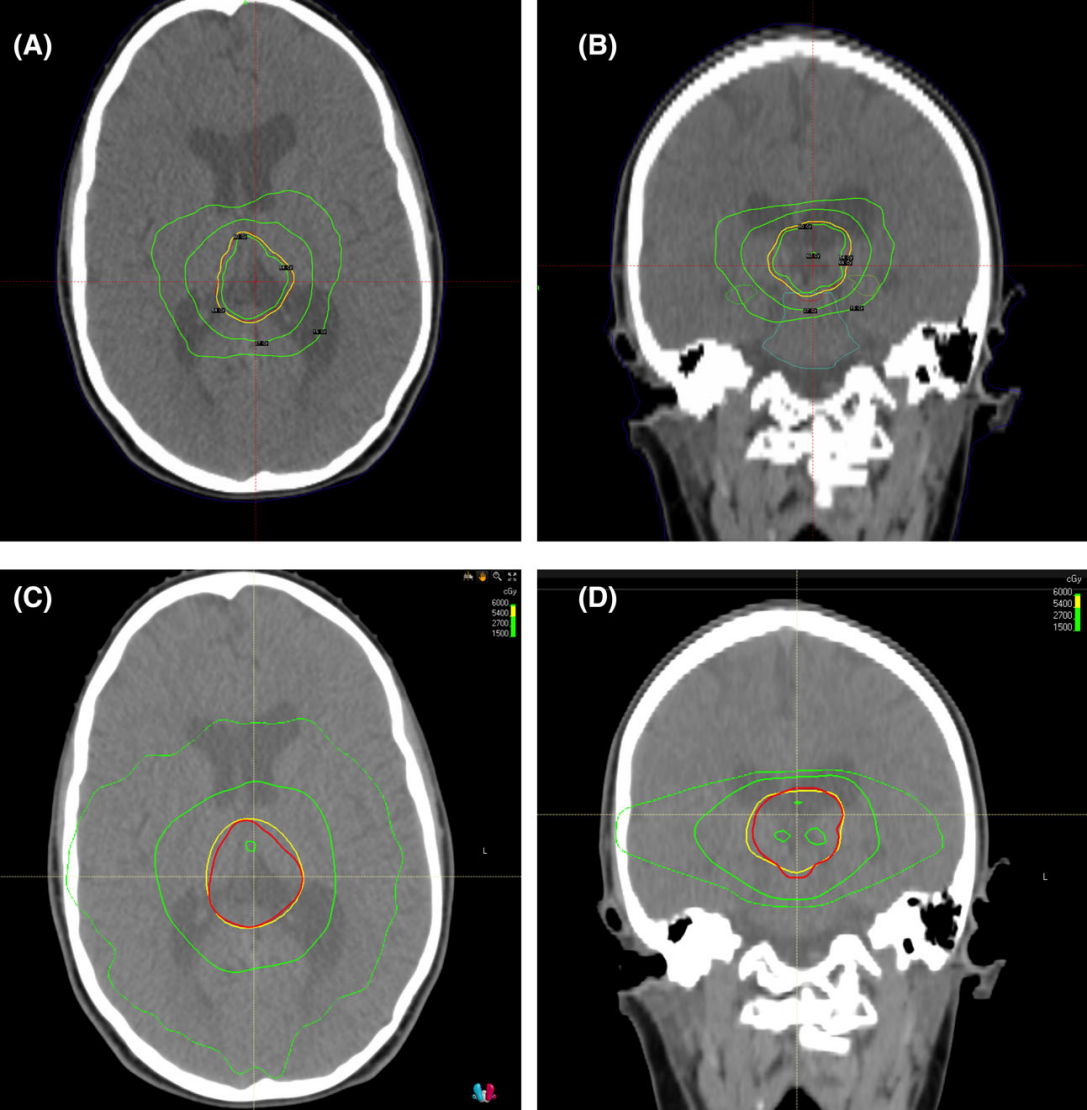
Visual comparison of the dose distributions for a single representative patient. Axial and coronal views of mcfSRT (panels A and B) and VMAT (panels C and D) dose distributions are shown. The PTV is represented with a red line. The innermost green line represents the 60 Gy isodose line. The yellow line represents the 54 Gy isodose line (prescription). The two outer lines represent the 27 and 15 Gy isodose lines. Note how the 15 Gy isodose line is more conformal to the target with mcfSRT, as compared to VMAT.

The median of the maximal doses for some OARs were lower in the mcfSRT plans (Table 3), for example the right optic nerve (1960 cGy vs. 4807 cGy, *P* = 0.014). In addition, the median doses to 0.1 cc for optic chiasm (2900 vs. 4743 cGy, *P* = 0.014) were also lower for mcfSRT compared to VMAT. The D50 for hippocampi were similarly less in mcfSRT plans compared with VMAT plans across each of the metrics evaluated (median Dmax, D0.1cc and D50; all comparisons *P*‐value < 0.05). The mcfSRT plans were noted to deliver as much as 40% less dose to these structures compared to VMAT technique, when comparing D50. A visual comparison of mcfSRT and VMAT dose distributions is shown in Figure 3.

## Discussion

The results of this study confirm our hypothesis that radiation planning done using a fractionated stereotactic radiotherapy technique with the mcfSRT system is dosimetrically feasible, fulfilling our a priori criteria of conformity index near 1 and sufficient PTV coverage. The median mcfSRT dose gradient index of 3.3 was slightly above our a priori threshold but remains dosimetrically feasible to deliver. Our data demonstrate that mcfSRT plans have similar or better target volume coverage as compared to VMAT plans. Thus, for well‐demarcated, non‐infiltrating tumours such as those selected for this dosimetry study, we believe that the mcfSRT plans would have equivalent efficacy compared to VMAT plans in tumour control based on similar prescription dose coverage.

The mcfSRT technique was consistently shown to deliver less radiation to organs at risk. The lower maximal dose to some OARs in mcfSRT plans is particularly advantageous for the protection of serial organs where maximum doses should be kept within tolerance for the preservation of function. Maximum dose to the brain must be carefully monitored and controlled during mcfSRT planning.

The dose gradient index is useful to compare treatment plans of equal conformity. The steep dose gradient outside the radiosurgical target is one of the factors that makes radiosurgery advantageous, allowing a higher dose to be delivered to the target, while minimising radiation exposure to the OARs. Gradient index can be used to compare different plans, explore optimal prescription isodoses or compare treatment modalities.^11^ The mcfSRT plans had a higher median conformity index and lower gradient index compared to VMAT plans, both attractive attributes in favour of mcfSRT planning. The higher conformity index indicated that the volume of the prescription isodose was more tightly shaped to the PTV in mcfSRT plans compared to VMAT plans, whereas the lower median gradient index for mcfSRT plans indicated steeper dose drop off. The physical and dosimetric advantages of mcfSRT allowed plans to deliver less radiation doses to the surrounding OARs across the reported metrics, including maximum dose, dose to 0.1 cc of structure as well as the median dose (D50). Thus, mcfSRT plans reduced radiation doses to the surrounding OARs better than VMAT plans. This is especially advantageous for tumours located close or adjacent to sensitive structures and potentially reduces the risks of long term radiotherapy toxicities.

Although higher conformity index may be advantageous for the preservation of OARs, it may actually be disadvantageous in tumour control. In a study by Aiyama et al.^12^ evaluating radiosurgery (Gamma Knife) for brain metastases, high conformity index, among other variables, was associated with a greater risk of local progression; cumulative incidences of local progression were significantly lower in patients with CIs <0.65. This is in contrast to the conventional opinion that radiotherapy plans with higher CI and lower GI result are superior, resulting in low local progression rates and minimal complications. However, in our study, our targets had a CTV contoured; in addition, we applied a 1 mm PTV to the mcfSRT plans, which should guard against geographic miss during highly conformal treatment. New indices of plan quality, beyond conformity index (which is dependent on target volume), are needed to ensure target coverage and reliable plan quality evaluation.

Interestingly, also from Aiyama's paper, a higher GI was not associated with increased toxicity.^12^ This may actually illustrate the importance of respecting the dose limitations of individual OARs, and that as long as usual dose constraints are observed, variations in dose gradient index do not provide further protections to the OARs. In addition, gradient index is strongly associated with target volume, which may have confounded findings. Nonetheless, plans with steep dose gradients allow for better preservation of OARs especially when the OARs are located close to the targets, and in younger patients for whom avoidance of late effects is of paramount importance.

Our findings are consistent with a study by Gavaert et al., which compared different radiosurgery equipment (CyberKnife, Perfexion and Novalis) in the treatment of arteriovenous malformations and acoustic neuromas.^13^ The study showed that single‐fraction SRS (Gamma Knife/Perfexion) can achieve a high degree of conformity with limited low doses surrounding the inhomogeneous dose distribution within the tumour, at the cost of increased treatment time. On the other hand, plans made using the dynamic conformal arc technique (Novalis) have improved dose homogeneity in the tumour and reduced treatment time, at the cost of conformity.^4^ However, our study is unique because we evaluated a novel mcfSRT system and demonstrated dosimetric feasibility in the setting of conventionally fractionated treatment. Prior reports of using the Leksell Gamma Knife® Icon™ for fractionated radiotherapy have been limited to short courses of RT over 5 fractions or less;^14^, ^15^, ^16^, ^17^ there are no known published reports of treatment courses over prolonged fractionation schedules using this device, though retrospective work to evaluate such treatments is ongoing (Dr Michael Yan, 2022, personal communication).

The characteristics of SRS plans that produce dosimetrically superior plans could be especially advantageous in the paediatric age groups where reduction of long term toxicities from radiation could improve a patient's quality of life as an adult. We can also potentially use this advantage in tumours that are located close to sensitive structures such as visual and auditory organs at risk.

There are a few limitations in this study including small number of cases and heterogeneous histologies at different intracranial sites. We specifically only included patients with well‐demarcated, non‐infiltrating tumours. Even though mcfSRT is delivered with a nominal 1.8–2 Gy/day, the maximal plan dose is higher than with VMAT, due to the heterogeneous dose distributions inherent to mcfSRT. Patients with infiltrating glial tumours (such as astrocytoma or oligodendroglioma) are likely less suitable for mcfSRT due to an increased risk of adverse radiation effect or radionecrosis in brain, especially with the larger treatment volumes required for these types of neoplasms.

## Conclusions

This is a preliminary, in silico dosimetry study which showed that mcfSRT planning is dosimetrically feasible to use in fractionated radiotherapy for intracranial tumours, with possible added advantage of better preservation of OARs. Based on our data, mcfSRT may improve OAR sparing over conventional, linear accelerator photon treatment. Further studies are needed to prospectively evaluate the feasibility and safety of treating selected patients using mcfSRT and to compare the treatment outcome with patients being treated on conventional linear accelerators.

## Funding

CHF was partially funded by the Princess Margaret Cancer Foundation. ZAL was funded by the Department of Radiation Oncology Academic Enrichment Fund.

## Conflict of Interest

The authors declare no conflict of interest.

## Ethics Approval

This retrospective, minimal risk study was reviewed by the University Health Network Research Ethics Board (17‐6280), with waiver of consent.

## Data Availability

The data are available upon request to corresponding author.
